# Rapid Calibration of Nanoliter per Second Flow Rate by Image Processing Technology

**DOI:** 10.3390/mi14061189

**Published:** 2023-06-02

**Authors:** Jiawei Luo, Cheng Yang, Yan Shen

**Affiliations:** 1 School of Aeronautics and Astronautics, Sun Yat-sen University (Shenzhen Campus), Shenzhen 518107, China; luojw33@mail2.sysu.edu.cn (J.L.);; 2 Shenzhen Key Laboratory of Intelligent Microsatellite Constellation, Shenzhen 518107, China

**Keywords:** microflow, image processing technology, rapid calibration, gravimetric method

## Abstract

The need for high-precision microflow control is increasingly evident across various fields. For instance, microsatellites employed in gravitational wave detection require flow supply systems with a high accuracy of up to 0.1 nL/s to achieve on-orbit attitude control and orbit control. However, conventional flow sensors are unable to provide the necessary precision in the nanoliter per second range, and thus, alternative methods are required. In this study, we propose the use of image processing technology for rapid microflow calibration. Our method involves capturing images of the droplets at the outlet of the flow supply system to rapidly obtain the flow rate, and we used the gravimetric method to verify the accuracy of our approach. We conducted several microflow calibration experiments within the 1.5 nL/s range and demonstrated that image processing technology can achieve the desired accuracy of 0.1 nL/s while saving more than two-thirds of the time required to obtain the flow rate within an acceptable margin of error compared to the gravimetric method. Our study presents an efficient and innovative approach to addressing the challenges of measuring microflows with high precision, particularly in the nanoliter per second range, and has the potential for widespread applications in various fields.

## 1. Introduction

The high-precision control of microflow has become increasingly important in various fields, including biomedical engineering, semiconductors, and microsatellites [1]. Specifically, microsatellites are subject to complex environmental distribution forces on orbit, such as solar radiation forces, which are constantly changing in the micro-Newton range [2]. To achieve the high-precision on-orbit attitude control and orbit control requirements for microsatellites, the propulsion system must compensate for non-conservative forces [3,4]. Among electric propulsion technologies, field emission electric propulsion has a specific impulse between 2000 s and 3000 s and possesses the characteristics of small thrust and a wide range of precise adjustment [4,5]. The thrust formula, $F=\dot{m}v$, highlights the need for a precision flow supply system with an accuracy of 0.1 nanoliters per second to achieve micro-Newton thrust control. Furthermore, the development of a flow supply system with an accuracy of 0.1 nL/s relies on the availability of a means to measure flow in the nanoliter per second range.

However, traditional flow sensors face limitations in achieving precision measurement in the nanoliter per second range [6] due to size effects and the limitations of microtubes [7,8]. Currently, microflow measurement methods can be divided into contact and non-contact techniques. Non-contact methods, such as the gravimetric method [9] and the volumetric method, can measure flow in the nanoliter range using an electronic balance with an accuracy of 0.1 mg, but they are time-consuming and suffer from liquid evaporation [10]. Moreover, these methods only reflect the average flow rate over a period of time, which is not conducive to rapid calibration of the nanoliter per second flow rate. The micro-PIV technique can construct a 3-D velocity field for flow rate measurement of 2.481 μL/min ~ 5.788 μL/min within a 4% relative error [11]. However, tracer particles used in this technique would contaminate the liquid, and microtubes used in the flow control system must be transparent [12]. Relying on the rapid development of MEMS technology, contact measurement has developed many types of flow sensors based on different measurement principles, such as thermal flow sensors, Coriolis mass flowmeters, differential pressure flow sensors, etc. Among them, thermal flow sensors have excellent measurement range and sensitivity but can only achieve a minimum measurable flow rate of 1 μL/min, and therefore cannot measure flow rates in the nanoliter per second range [13]. Flow sensors, based on a unique measurement principle, cannot measure flow rates in the nanoliter per second range. To achieve smaller flow rate measurements, Joost C. Lötters utilized micromachining technology to integrate a thermal flow sensor and micro-Coriolis mass flowmeter on a single chip, achieving a wide range and high accuracy in flow measurement from 100 nL/h to 10 mL/h. However, the integrated flow sensor system chip is still in the experimental stage and cannot be widely used for flow measurement in the nanoliter per second range due to the uncertainty of the measurement scenario and the limitations of the pipe joint, as it is a contact measurement sensor [14].

In this study, we present a novel approach for rapid calibration of nanoliter per second flow rates in microfluidic systems utilizing image processing technology, based on the flow supply system built with the expectation of controlling flow rate with a resolution of 0.1 nL/s. The high resolution of an HD camera is leveraged to calibrate the flow rate frame-by-frame, which enables precise and efficient measurements. This approach is distinct from liquid level measurement [15], as it calibrates the flow rate of the system by measuring the drop at the outlet, thus reducing the requirements on the flow supply system pipe and measurement environment. In comparison to conventional methods, such as traditional flow sensors, non-contact techniques, and contact measurement methods, the proposed approach overcomes limitations associated with achieving precision in the nanoliter per second range. The micro-PIV technique, which requires transparent microtubes and may lead to liquid contamination, is also surpassed. By leveraging image processing technology and high-resolution cameras, the proposed method enables accurate frame-by-frame calibration and reduces the constraints on the flow supply system and measurement environment.

To validate the effectiveness of our proposed method, we conducted experiments using both the gravimetric method and image processing technology to calibrate the flow rate of the flow supply system, which adjusts flow rate by changing the pressure differential. The results demonstrate the feasibility of using image processing technology for the rapid calibration of nanoliter per second flow rates in microfluidic systems. Overall, this study provides a promising approach for enhancing the precision and efficiency of microflow measurements, with potential applications in a wide range of fields.

## 2. Experimental System

### 2.1. Flow Supply System and Liquid

**Figure 1:**
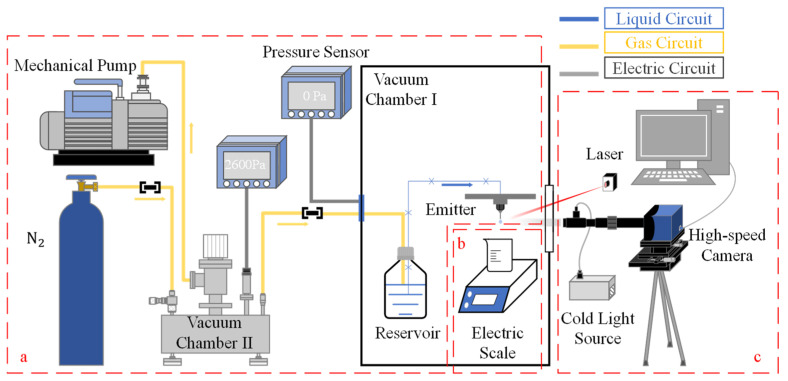
General diagram of the experimental setup: (**a**) vacuum pressure differential microflow supply system; (**b**) precision electronic weighing system; (**c**) image acquisition system.

In this study, we have developed a flow supply system comprising a liquid reservoir, liquid conveying pipe, emitter, vacuum chamber, high-pressure gas holder, and mechanical pump, as depicted schematically in Figure 1a. The flow rate is controlled by varying the pressure differential between the reservoir and the outlet of the emitter. Dry nitrogen is used to pressurize the fluid reservoir. The three main components, i.e., the fluid reservoir, liquid conveying pipe, and emitter, are placed in vacuum chamber Ⅰ, with the fluid reservoir connected to vacuum chamber II via a gas conveying pipe. During system operation, vacuum chamber I is maintained at a high vacuum, while the pressure of vacuum chamber II is adjusted to modify the pressure differential between the fluid reservoir and the environment to which the emitter is exposed.

**Figure 2:**
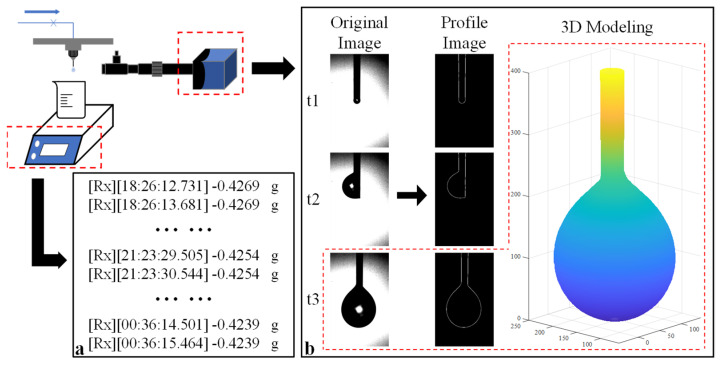
Schematic illustration of the measurement concepts: (**a**) measurement concept utilizing the gravimetric method; (**b**) measurement concept utilizing image processing technology. The red square indicates the 3D volume model obtained from the original image (t = t3) and corresponding profile image.

When the pressure differential between vacuum chambers Ⅰ and Ⅱ reached the appropriate value, both measurement systems were initiated to record data. A detailed illustration of the measurement concept is presented in Figure 2. The electric scale captured weight data at a frequency of 1 Hz, as demonstrated in Figure 2a. Simultaneously, the HD camera captured original images, which were subsequently processed to generate profile images. Utilizing the axisymmetric droplet model, the 3D volume of the droplet was calculated, as shown in Figure 2b. This calculation method will be further elucidated in Section 2.3. By analyzing the variation in volume over time, the flow rate can be accurately determined.

We employed an ionic liquid propellant, namely EMI-Im, and its relevant liquid properties are presented in Table 1 [16]. As is widely known, ionic liquids possess a remarkably low vapor pressure, implying that the formation of bubbles hindering the transport of liquid in the micropipe is highly unlikely in a low-pressure environment. To further prevent the formation of bubbles, we degassed EMI-Im by vacuum-pumping before experimentation and conducted multiple tests on various pipeline designs to determine the appropriate material and inner diameter size of the liquid conveying pipe. Relevant pipeline information is presented in Table 2.

Ignoring the fraction of the inner surface of the pipe, the flow rate through the emitter can be described by a Poiseuille-type relation [17]:

(1) $$Q=\frac{\pi R_{i}^{4}}{8\mu L}\left(\Delta P-\rho gh\right)$$

where Q is the volumetric flow rate, $R_{i}$ is the emitter inner radius, μ is the viscosity of the liquid, L is the length of the fluid conveying pipe and emitter, ΔP is the pressure differential between the liquid reservoir and the vacuum chamber Ⅰ, and h is the vertical distance between the liquid level and the tip of emitter. The vacuum chamber Ⅰ employed in this study can maintain a high vacuum pressure of $1\times 10^{-4}$ Pa, which is negligible compared to the liquid reservoir pressure range of $1000\;Pa\sim 10^{4}\;Pa$. As a result, the pressure differential (ΔP) is primarily influenced by the liquid reservoir pressure, which can be finely tuned with a resolution of 1 Pa.

In addition to pressure losses resulting from frictional forces along the pipeline, area changes in the cross-section due to pipeline connections can also lead to local pressure losses. Local resistance is defined as the resistance arising from the impact of the fluid with the wall and the collision between fluid masses, which causes local loss, defined as the energy loss per unit weight of fluid. According to the empirical formula, the local loss can be calculated:

(2) $$h_{f}=\zeta_{1}\frac{v_{1}^{2}}{2g}=\zeta_{2}\frac{v_{2}^{2}}{2g}$$

where $\zeta_{1}$ is the local pressure coefficient for the expanding pipe, and $\zeta_{2}$ is the local pressure coefficient for the narrowing pipe. Converting to a pressure loss, the expression is

(3) $$p_{f}=\zeta_{1}\frac{{\rho v}_{1}^{2}}{2}=\zeta_{2}\frac{{\rho v}_{2}^{2}}{2}$$

and the local pressure coefficient is calculated by Equation (4):

(4) $$\zeta_{1}={\left(1-\frac{A_{1}}{A_{2}}\right)}^{2}\;\;\zeta_{2}=0.5\times \left(1-\frac{A_{2}}{A_{1}}\right)$$

According to Equations (3) and (4), the local pressure loss due to the area changes in cross-section of the pipe used in this experiment in the flow range of 0 to 10 nL/s is calculated to be $\leq 2.0517\times 10^{-4}$ Pa, which is much smaller than the adjustable pressure accuracy of 1 Pa and the pressure loss along the pipeline due to the nanoliter flow rate. Therefore, it can be considered that the local pressure loss caused by the area changes of the pipeline cross-section is negligible.

Consequently, in this study, the pipeline design has been confirmed and the local pressure loss is negligible; thus, we can introduce the hydraulic resistance to describe the relationship between the flow rate and the liquid reservoir pressure:

(5) $$Q=\frac{\left(\Delta P-\rho gh\right)}{R_{h}}$$

where $R_{h}$ is the hydraulic impedance of the pipeline, which is a fixed value for the pipeline used in this article. Additionally, the relationship between pressure differential and volumetric flow rate can be described as

(6) ΔP=4083×Q+2232

In accordance with Equation (6), a pressure differential of approximately 408.3 Pa corresponds to a flow rate differential of 0.1 nanoliters per second, indicating that the flow supply system can ideally achieve flow control with a resolution of 0.1 nanoliters per second.

### 2.2. Gravimetric Method

In this study, we utilized the gravimetric method to investigate the feasibility of using image processing technology for rapid microflow calibration. Specifically, droplets generated from the emitter tip were collected in a small beaker, and their weight was measured using an FA3204C Electronic Scale, which has a scale range of 320 g, reading accuracy of 0.1 mg, and a typical weighing time of 5–8 s. The electronic scale was located in vacuum chamber I, while a beaker was placed under the pendant emitter to collect the droplets, as shown schematically in Figure 1b. After the pressure of the vacuum chamber Ⅱ was adjusted to the target value and the first drop had fallen, we started to record the data as part of the formal experiment. The droplet weight was recorded on a PC for subsequent analysis. To minimize measurement errors caused by liquid remaining on the emitter or being dragged from the emitter by the falling droplet, we updated the measured average flow rate by summing the weight of multiple droplets while ensuring that the pressure differential (ΔP) remained within an acceptable error range, as described by Equation (3):

(7) $${\left.Q_{j}\right|}_{\Delta P}=\frac{\sum_{i=1}^{j}m_{i}}{\rho \sum_{i=1}^{j}t_{i}},i=1,2,\ldots ,j$$

### 2.3. Image Processing Technology

The equipment used for image processing technology was divided into two parts: optical equipment and a PC for processing droplet images, as depicted in Figure 1c. The optical equipment comprised a camera, lens, adapters, and an optical source. The MotionBLITZ EoSens^®^ mini1 was chosen due to its high image resolution of 1280 [H] × 1024 [V] and 8-bit monochrome, which meets the requirement for high image resolution. The camera’s Gigabit Ethernet interface facilitated the operation and recording of image data on a PC. We selected the POMEAS VP-LZL-12101D zoom lens, which enables coaxial light incidence with a double magnification adapter. An infrared light source with oblique forward incidence was chosen as the optical source.

Before the formal experiment began, the coarse adjustment was made to enable the shooting distance from the lens to the emitter to meet the working distance of the lens and make the emitter image appear in the field of the lens’ view. The position and intensity of the light source were adjusted to improve imaging clarity. The camera shooting distance was further adjusted by the XYZ linear translation stage, the object distance was adjusted to achieve focus, and the image was adjusted to the appropriate position. After the preparation of the optical components, the vacuum chamber Ⅰ can be closed and the pumping can begin. Upon achieving a stable flow, we recorded real-time images of the droplets and employed MATLAB to extract their edges.

**Figure 3:**
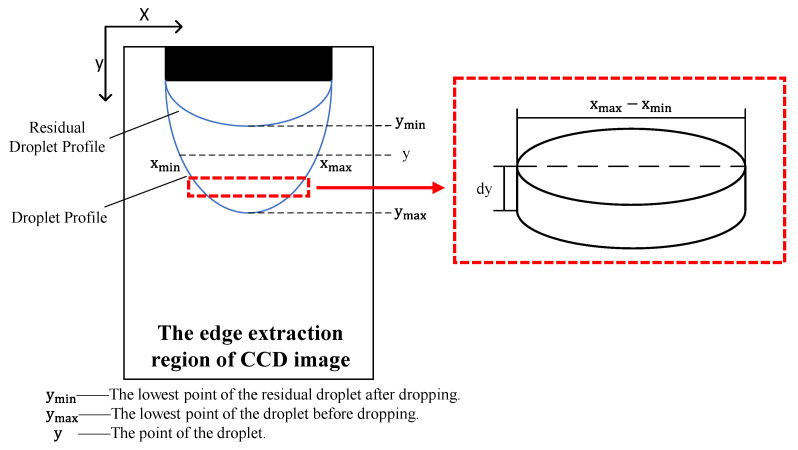
Diagram of the droplet volume calculation.

Given that the emitter was mounted nearly vertically, the droplet was assumed to be pendant and axisymmetric [15,18]. Using this axisymmetric model, we split the droplet into a collection of cylindrical volume elements along the vertical direction [19], as shown in Figure 3. We integrated the droplet volume element along the direction of droplet growth to determine the pixel volume of the droplet. To confirm the proportional relationship between the pixel and the real size, we defined a proportion coefficient as the ratio of the outer diameter of the emitter to the corresponding pixel, as expressed in Equation (8). This allowed us to calculate the real volume of the droplet from the pixel volume, as shown in Equation (9).

(8) $$D={\left[\frac{the\;outer\;diameter\;of\;the\;emitter\left(mm\right)}{the\;outer\;diameter\;pixel\;of\;the\;emitter}\right]}^{3}$$

(9) $$Vi=D\int_{y_{min}}^{y_{i}}\frac{{\left(x_{max}-x_{min}\right)}^{2}\pi }{4}dy$$

## 3. Results and Discussion

In this paper, we set up and completed five sets of flow rate calibration experiments at different pressure differentials: 2600 Pa, 3000 Pa, 4000 Pa, 6000 Pa, and 8000 Pa. These experiments were carried out simultaneously by the mass method and image processing technology. Additionally, each set included at least three droplet generation cycles. In this section, we will first analyze each set of experiments to ensure that the flow rates were in the nanoliter range and met the flow control design expectations. Then, we will use the results from the gravimetric method to verify the feasibility of using image processing technology to achieve the rapid calibration of the nanoliter per second flow rate.

### 3.1. Results of the Gravimetric Method

Using the gravimetric method, we recorded the weight of each droplet and the time it took to accumulate. The specific data are shown in Table 3, indicating that the weight of each droplet at different pressure differentials is 1.5 mg. Based on the balance between surface tension and gravity, as expressed in Equation (10), the theoretical weight of the droplet under the specific pipeline design is 1.4961 mg when the fluid is stationary. The deviation between the ideal weight and the experimental value is 0.56%. Considering that the resolution of the electronic balance used in the experiment is 0.1 mg, this deviation can be disregarded. Hence, we can conclude that when the flow rate is stable and less than 1.44 nL/s, the equilibrium between surface tension and gravity is applicable, and the weight of the droplet can be calculated by surface tension.

(10) Mg=2πRγ

**Figure 4:**
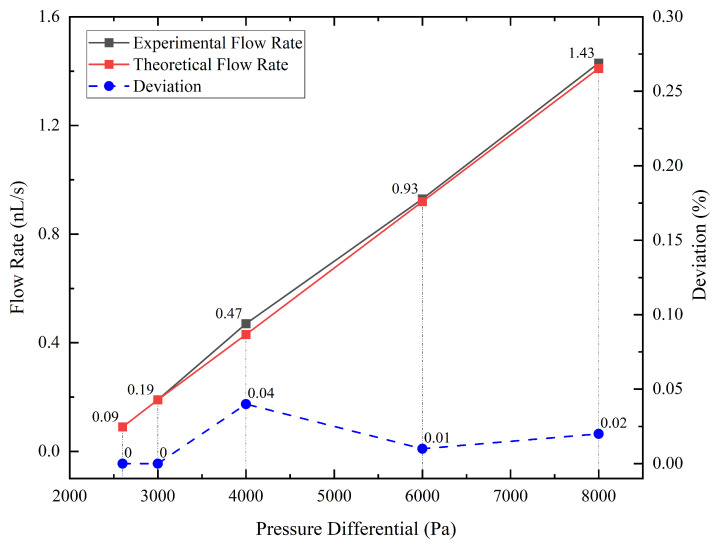
Comparison of experimental flow rate, theoretical flow rate, and deviation at various pressure differential.

After we obtained the data about the weight of droplets and the time it took them to accumulate, we used Equation (7) to calculate the experimental value of the average flow rate at different pressure differentials and Equation (5) to calculate the corresponding theoretical flow rate. As shown in Figure 4, the red line is the theoretical value, and the black line is the experimental value, which is very close to the red line and almost linear. Additionally, the blue dotted line is the deviation, which is the experimental value minus the theoretical value. The maximum error is 0.037 nL/s, which is less than the 0.1 nL/s anticipated resolution of the flow supply system, meaning that the flow supply system achieves the design expectations.

Considering that the maximum deviation occurred in the set of the 4000 Pa pressure differential, we must take into account two additional sources of error that may affect the accuracy of our measurements. First, the electronic balance used in the experiment has a resolution of 0.1 mg, which limits the precision of our weight measurements. Second, some degree of leakage may occur in the vacuum chamber, which could affect the pressure differential and the flow rate. However, we believe that the impact of these factors is minimal, given that the deviation between the theoretical and experimental values is within an acceptable range. Based on the experimental results, the flow control system was able to adjust the flow rate with a resolution of 0.1 nL/s, as anticipated. Moreover, the theoretical value or the linear fit to the experimental value can be used to predict the flow rate of the flow control system at a given pressure differential. Hence, the result confirms the validity of the experimental setup and provides a reliable basis for subsequent analyses.

### 3.2. Results of Image Processing Technology

**Figure 5:**
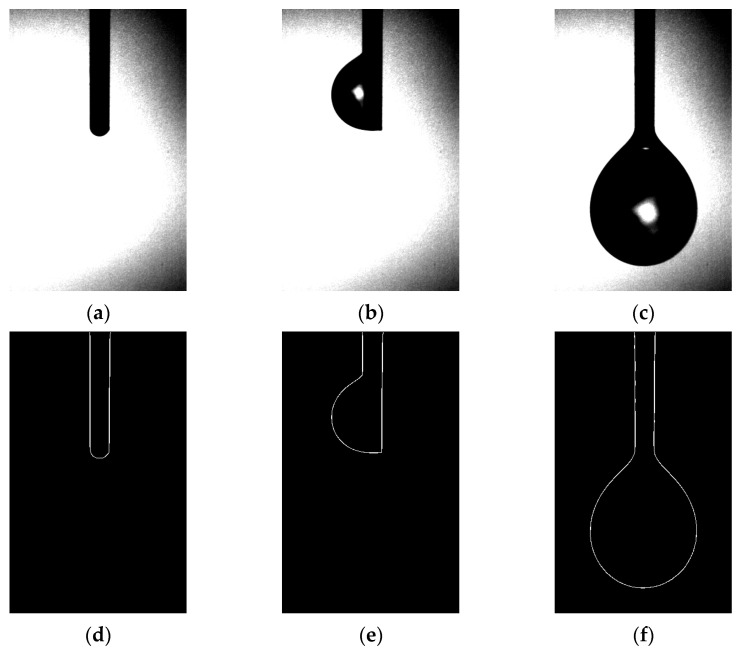
Droplet images and extraction edges: (**a**–**c**) diagram of the droplet growth process under the vertical emitter; (**d**–**f**) diagram of the contour of the corresponding images.

Section 3.1 of our study detailed the gravimetric method employed for droplet analysis, while Section 3.2 introduced a novel approach using image processing technology for droplet measurement. High-speed imaging was employed to capture the droplet growth process, as demonstrated in Figure 5. The images clearly revealed that during droplet growth, the liquid emerging from the emitter outlet accumulated at different locations, ranging from the emitter needle port to the emitter sidewall, before sliding down to the emitter needle port for accumulation until droplet detachment. Based on this observation, we divided the droplet growth process into three stages, corresponding to Figure 5a–c: (i) formation of a liquid film at the emitter needle port; (ii) droplet attachment on the emitter sidewall; and (iii) droplet detachment and further accumulation at the emitter needle port. However, it is noteworthy that in an ideal scenario, liquid flow from the emitter needle port should not infiltrate upwards along the emitter outer wall and accumulate on one side. Considering the size effect of the emitter, we propose three assumptions: (i) the emitter is not fixed vertically along the direction of gravity, resulting in liquid outflow on the emitter side with a small wetting angle and upward infiltration of the emitter along the outer wall, which continues to pull subsequent outflow until the resistance along the emitter’s outer wall can no longer offset the accumulated liquid’s own gravity, leading to downward sliding towards the emitter needle port; (ii) the emitter needle port is not flat, and there exists a fine gap for liquid infiltration; (iii) prior to the formal experiment, the liquid flow rate in the pipeline was initially set at a relatively high rate (>5 nL/s) and then subsequently reduced to a lower flow rate (<1 nL/s). As a result, liquid backflow occurred at the emitter needle port, leading to the initial droplet accumulation at the needle mouth. This accumulation contaminated the outer wall of the emitter, facilitating liquid infiltration.

After obtaining high-speed camera images of the droplet growth process (as displayed in Figure 5), image segmentation and threshold separation operations are performed to identify and extract droplet contours using the Canny–Sobel. The extracted contours enable precise droplet volume calculations, as shown in Figure 5d–f. However, the extent of droplet infiltration on the emitter outer wall during the second stage of droplet growth is not entirely reflected by one-dimensional imaging and gray value processing. This is because assuming that the uninfiltrated side of the emitter outer wall always serves as the droplet boundary in axisymmetric droplet modeling can lead to a calculated volume larger than the actual volume. Despite yielding an experimental value greater than the actual value, this error gradually reduces as droplets continue to accumulate and increasingly conform to the axisymmetric droplet model.

In this experiment, the outer diameter of the emitter, with a diameter of 0.25 mm, was obtained by employing image processing techniques, yielding an outline of 41 pixels. Based on Equation (4), the scale factor D for this experiment was calculated as 0.0061 mm/pixel. With this factor, we were able to compute the volume of each droplet from the droplet image frames. To obtain the droplet volume for comparison with the volume obtained through the gravimetric method, we utilized the image processing method to obtain the droplet volume prior to the droplet falling and subsequently subtracted the droplet volume after falling. Due to the limitations of the image memory, every 1635 shots must be saved, and the image data is lost during the image storage period. If the droplet falls during this period, the mass data of the falling droplet under the image method cannot be obtained. Therefore, to increase the control sample between the gravimetric method and the image processing technology method in terms of droplet mass measurement, electronic balance mass records and images were retained after each pressure differential grouping experiment (without controlling for pressure differential changes due to air leakage), as shown in Table 4.

Table 4 shows that the droplet masses acquired through image processing are in agreement with those obtained by the mass method. The maximum relative error is only 3.15%, which can be attributed to the image vibration noise and the measurement accuracy of the electronic balance. It should be noted that in the context of microflow measurement, the error introduced by mass measurement is substantially smaller than the required accuracy for flow measurement. Therefore, we can confidently assert that the droplet volume and mass data derived from high-resolution camera imaging, image processing techniques, and the axisymmetric droplet model are highly reliable.

**Figure 6:**
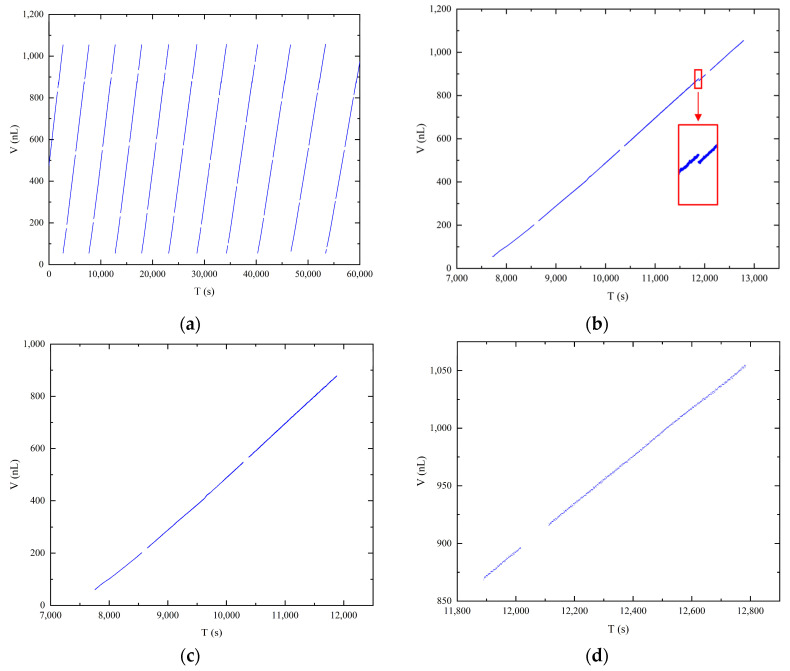
Correlation between time and droplet volume: (**a**) full experimental time record; (**b**) one cycle of the droplet growth period; (**c**) first and second stages of the droplet growth process; (**d**) third stage of the droplet growth process.

Figure 6a displays the volume data obtained from image measurements at a pressure differential of 4000 Pa. Each straight line in the graph represents a distinct droplet growth period, encompassing nine relatively complete droplet growth periods. It is important to note that due to the attachment of liquid to the emitter sidewall during the droplet growth period, it was not possible to intercept the emitter completely in each image. Consequently, the volume of the emitter exposed in the image was calculated as the initial value for each line. The presence of gaps between the discrete data points represents the loss of data during the image saving period, as mentioned previously. Despite these gaps, a clear linear correlation between droplet volume and time is observed, as depicted in the scatter plot of the image volume data. For detailed analysis, a single cycle of droplet growth data was selected, as illustrated in Figure 6b. A distinct break point, indicated by the red box, is observed on the graph. This break point corresponds to the accumulation of droplets slipping from the emitter sidewall to the emitter needle port, aligning with the three-stage droplet growth process described in Section 3.2. As the first stage provides a limited number of samples at a shooting rate of 1 frame per second, resulting in approximately 10 data points at the set pressure differential of 4000 Pa, it is not suitable for individual data analysis. Therefore, the data from the first and second stages were combined, as shown in Figure 6c, while the third stage data are plotted separately in Figure 6d. A linear fit was performed on the data at both ends to obtain the corresponding slope as the average flow rate. The slopes obtained for both sets of data are 0.4818 and 0.4768, which are similar to the flow rate of 0.47 nL/s obtained by the gravimetric method. The fitted flow rate obtained in the second stage is greater, confirming the analysis of the source of error in the second stage calculation. Using the flow rate obtained by the gravimetric method as the true value, the error in the larger flow rate obtained by fitting the data obtained by the image method in the second stage is only 2.5%. This result demonstrates the feasibility of calibrating microflows using the image method. The high accuracy of the image processing technique for droplet images based on the axisymmetric droplet model, as described in Section 2.3, further supports the reliability of the obtained droplet volume and mass data.

**Figure 7:**
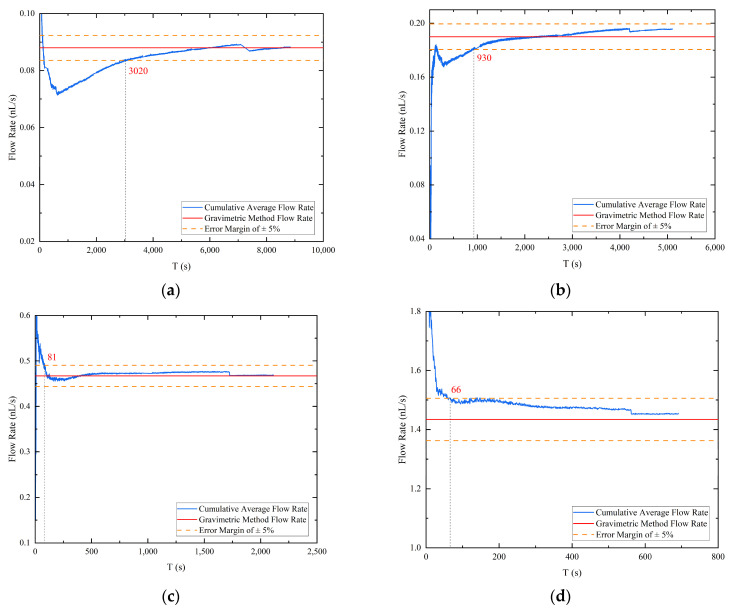
The accumulation average flow rate: (**a**) pressure differential 2600 Pa; (**b**) pressure differential 3000 Pa; (**c**) pressure differential 4000 Pa; (**d**) pressure differential 8000 Pa.

In order to expedite flow calibration, a cumulative average flow rate was computed from the droplet volume acquired through image recognition, leading to a cumulative flow curve as a function of time for four different pressure differentials, as depicted in Figure 7. The blue line represents the cumulative average flow rate curve, the red line denotes the flow rate value obtained by the gravimetric method corresponding to the pressure differential, and the orange dotted line represents the gravimetric method flow rate value with an error margin of plus or minus 5%. For the experiment conducted at a pressure differential of 2600 Pa, the cumulative average flow rate calculated by image processing required only 26.3% of the droplet drop time to converge to the “true flow rate”, while the other three groups required 18.2%, 3.8%, and 9.5% of the droplet drop time, respectively, at their respective pressure differentials. In contrast to the gravimetric method, which calculates the stable flow by weighing the dripping liquid, the image processing method can save more than two-thirds of the time by accumulating the average flow. Therefore, assuming a stable flow, the cumulative flow rate obtained by the image processing technique can converge to within plus or minus 5% of the actual flow rate obtained by the mass method in significantly less time than the droplet generation time. This approach demonstrates a considerable improvement in the speed of nanoliter per second flow calibration and highlights the potential for using image processing techniques to obtain accurate flow measurements.

During the high hold state of the vacuum chamber, the operation of the pump set generates vibrations that can cause shaking of the droplet image. This, in turn, can result in errors in the extraction of boundary contours, typically at the single-digit pixel level. Particularly at low flow rates where the volume change rate of the droplets is extremely small, these errors can become comparable to the error induced by the vibration. To overcome this issue, the cumulative average flow is computed to characterize the flow at the outlet of the emitter. However, due to the one-dimensional imaging and vertical fixation of the emitter, an error arises between the actual droplet volume and the identified droplet volume in the first and second phases of droplet accumulation. Nevertheless, by computing the cumulative average flow rate, the error between the two is continuously reduced, resulting in a better characterization of the rate of change of the droplet volume (i.e., flow rate). As shown in Figure 7, the accumulation of droplets sliding from the emitter side to the emitter exit causes significant step changes in the late cumulative average flow calculation.

## 4. Conclusions

To verify the feasibility of using image processing techniques for the calibration of nanoliter per second flow rate, five sets of measurements using the gravimetric method and the image processing method were conducted simultaneously at different pressure differentials, with theoretical flow rates ranging from 0.09 nL/s to 1.41 nL/s. The results of the gravimetric method measurements demonstrated that the flow supply system used in this study has the capability to generate a nanoliter per second flow rate by adjusting the pressure differential between the large and small vacuum chambers. The maximum error between the cumulative average flow rate and the theoretical flow rate measured by the gravimetric method did not exceed 0.037 nL/s. The image processing method allowed for the extraction of droplet profiles and the division of the droplet growth process into three stages based on where the droplets accumulate. By comparing the droplet mass obtained by the gravimetric method of weighing with the corresponding droplet mass calculated by the image processing method of identification, the maximum relative error did not exceed 3.15%, demonstrating that the mass and volume of accumulated droplets at the emitter exit can be accurately obtained by the image processing method. To further realize the rapid calibration of the nanoliter per second flow rate, the cumulative average flow rate was calculated from the image processing method results based on the prerequisite assumption of a stable flow rate. The results showed that the cumulative average flow rate converged to within an error of ±5% of the actual flow rate value within 2/3 of the droplet drop time for different pressure differentials. This study concludes that identifying droplets at the outlet of the pipeline at microflow rates using image processing and calculating the cumulative average flow rate according to the flow rate calibration accuracy can achieve rapid calibration of a steady-state microflow control system.

While our study focused on a specific type of ionic liquid, the image processing technology employed in this research can be adapted and optimized to suit various fluid properties and characteristics. The underlying principles of using image processing technology to achieve rapid calibration of nanoliter per second flow rates remain applicable across different liquids. Moreover, the real-time flow rate measurement capability of the technique offers potential for its utilization as a feedback loop in flow control systems. By continuously monitoring and adjusting the pressure based on the measured flow rate, it becomes feasible to attain and sustain a constant flow. Further investigations should explore these possibilities and assess the viability of employing this technique as both a calibration system and a feedback control mechanism for achieving precise flow regulation.

## Figures and Tables

**Table 1:** Material properties for EMI-Im [16].

Property	Symbol	Value at 298 K
Density	ρ	1518.48 kg m^−3^
Viscosity	μ	0.03246 Pa s
Surface Tension	γ	0.0359 N m^−1^
Electrical Conductivity	κ	0.921 S m^−1^
Relative Permittivity	ε_r_	12.25

**Table 2:** The pipeline parameters.

Pipeline Design	Material	Type	Size
Liquid Conveying Pipe	Peek	Inner diameter	0.1 mm
		Length	300 mm
Emitter	Stainless steel	Inner diameter	0.13 mm
		Length	25 mm
Liquid Level High Difference *			150 mm

* Vertical distance from the liquid level to the tip of emitter.

**Table 3:** Data on droplet weight, time to accumulation and average volume flow at different pressure differentials by gravimetric method.

Pressure Differential	Mass of Droplet	Time	Average Volume Flow
2600 Pa	1.5 mg	10,643 s	0.09 nL/s
	1.5 mg	11,554 s	0.09 nL/s
	1.5 mg	11,505 s	0.09 nL/s
3000 Pa	1.5 mg	5062 s	0.20 nL/s
	1.5 mg	5108 s	0.19 nL/s
	1.5 mg	5206 s	0.19 nL/s
4000 Pa	1.5 mg	2093 s	0.47 nL/s
	1.5 mg	2114 s	0.47 nL/s
	1.5 mg	2137 s	0.46 nL/s
6000 Pa	1.5 mg	1051 s	0.94 nL/s
	1.5 mg	1061 s	0.93 nL/s
	1.5 mg	1072 s	0.92 nL/s
8000 Pa	1.5 mg	684 s	1.44 nL/s
	1.5 mg	689 s	1.43 nL/s
	1.5 mg	693 s	1.40 nL/s

**Table 4:** Data on droplet mass by the gravimetric method and image processing technology, and the relative error.

Pressure Differential	Mass of Droplet (the Gravimetric Method)	Mass of Droplet (the Image Processing Technology)	Relative Error
~2600 Pa	1.5 mg	1.516 mg	1.06%
~3000 Pa	1.5 mg	1.517 mg	1.13%
	1.5 mg	1.517 mg	1.14%
	1.5 mg	1.519 mg	1.23%
	1.5 mg	1.518 mg	1.22%
~4000 Pa	1.5 mg	1.503 mg	0.17%
	1.5 mg	1.505 mg	0.34%
	1.5 mg	1.504 mg	0.29%
	1.5 mg	1.506 mg	0.41%
~6000 Pa	1.5 mg	1.519 mg	1.22%
	1.5 mg	1.522 mg	1.45%
	1.5 mg	1.521 mg	1.30%
	1.5 mg	1.520 mg	1.44%
~8000 Pa	1.5 mg	1.527 mg	1.77%
	1.5 mg	1.529 mg	3.15%

## Data Availability

The data that support the findings of this study are available from the first author, [Jiawei Luo], upon reasonable request.
